# Infrared spectral data of natural and man-made textile fibres for material identification and classification

**DOI:** 10.1016/j.dib.2026.112594

**Published:** 2026-02-13

**Authors:** Reeha Parkar, Angelica Jain, Miranda Prendergast-Miller, Thomas Stanton, Kelly J. Sheridan, Matteo D. Gallidabino

**Affiliations:** a King’s Forensics, Department of Analytical, Environmental & Forensic Sciences, King’s College London, London, SE1 9NH, UK; b School of Geography and Natural Sciences, Northumbria University, Newcastle Upon Tyne, NE1 8ST, UK; c Department of Geography and Environment, Loughborough University, Loughborough, LE11 3TU, UK

**Keywords:** ATR-FTIR spectroscopy, Chemometrics, Polymer characterisation, Material authentication, Machine learning, Forensic analysis, Environmental studies

## Abstract

This article presents a structured dataset of attenuated total reflectance Fourier transform infrared (ATR-FTIR) spectra acquired from natural and man-made textile fibres, compiled to support research in forensic, analytical, and environmental science. The collection comprises 160 spectra obtained from 137 verified textile samples originating from multiple sources, including industry reference collections and academic textile archives. The dataset includes several processing levels: (1) raw instrument files in PerkinElmer .sp format; and (2–5) tabular feature matrices containing unprocessed transmission spectra, baseline-corrected spectra, averaged baseline-corrected spectra grouped by fibre subtype, and fully pre-processed spectra prepared for chemometric and machine-learning workflows. All spectra were recorded using a Frontier FT-IR spectrometer by PerkinElmer equipped with a single-reflection diamond ATR accessory. Measurements were performed over the 4000–550 cm⁻¹ range at 4 cm⁻¹ resolution with four co-added scans per acquisition. Metadata describing sample identity and fibre characteristics are supplied. The resulting collection provides an openly accessible resource for material identification, spectral comparison, and the development or benchmarking of classification algorithms.

Specifications Table

**Table:** 

Subject	Engineering & Materials science
Specific subject area	ATR-FTIR characterisation of textile fibres for forensic, materials, and environmental applications.
Type of data	*Raw instrument files (.sp);* *Exported feature tables, before and after pre-processing (.csv);* *Python script (.py).*
Data collection	Spectra were collected using a PerkinElmer Frontier FT-IR spectrometer equipped with a single-reflection diamond ATR accessory. Samples were analysed in transmission mode over the 4000–550 cm⁻¹ range at 4 cm⁻¹ resolution with four co-added scans. Multiple spectra were acquired per sample, and one or several high-quality measurements were retained. All spectra were acquired using PerkinElmer Spectrum software v10.4.00 and processed using Python v3.12.0.
Data source location	King’s Forensics, Department of Analytical, Environmental and Forensic Sciences, King’s College London, United Kingdom
Data accessibility	Repository name: Mendeley Data Data identification number: 10.17632/rx3fjgz96x.3 Direct URL to data: https://doi.org/10.17632/rx3fjgz96x.3 All files are openly available under a Creative Commons CC BY 4.0 licence and can be downloaded individually or as a complete dataset package.
Related research article	None

## 1 Value of the Data

• These data provide a comprehensive reference collection of infrared spectra from natural and man-made textile fibres, measured under consistent instrumental conditions. The dataset covers a wide variety of materials commonly found in clothing and household fabrics, offering a standardised foundation for fibre characterisation and comparison.
• The dataset can be reused to develop, benchmark, and validate algorithms for spectral matching, material classification, or fibre identification across forensic, analytical, and environmental science applications. It is suitable for a wide range of computational approaches, including chemometrics and machine-learning models.
• The inclusion of multiple processing levels supports varied analytical needs. Raw transmittance spectra allow users to apply their own workflows; baseline-corrected spectra serve as ready-to-use reference profiles for visual or manual identification; and fully pre-processed spectra are optimised for chemometric and machine-learning applications.
• The metadata accompanying each sample allow users to relate spectral features to material descriptors such as fibre class, type, and subtype. This supports extended applications such as building spectral libraries, studying variability within and between textile categories, and evaluating spectral features relevant to discrimination tasks.
• The different spectral pre-treatment pipelines implemented were evaluated using support vector machine (SVM) and random forest (RF) classifiers under cross-validation to enable objective comparison. The resulting performance metrics provide a reference baseline for future studies using comparable ATR-FTIR data and classification approaches.
• The dataset will benefit forensic laboratories involved in fibre examination, environmental scientists investigating textile microfibre pollution, materials scientists and polymer chemists engaged in material identification, and data scientists developing and benchmarking chemometric and machine-learning models. It also provides an open teaching resource for students and educators in spectroscopy, textile analysis, and applied computational modelling.

## 2 Background

The dataset was compiled to support research on the spectroscopic characterisation and classification of textile fibres using infrared techniques. Fibre identification is an essential task in both forensic and environmental science [[1], [2], [3], [4]], where analysts frequently need to determine material composition from small samples. Attenuated total reflectance Fourier transform infrared (ATR-FTIR) spectroscopy provides a rapid, non-destructive method for distinguishing between fibre types based on their polymer composition and characteristic absorption features [[5], [6], [7], [8]].

Existing reference collections are often limited in scope, heterogeneous in acquisition conditions, or not publicly accessible, hindering method validation and comparative studies. To address this gap, a consistent series of ATR-FTIR measurements was acquired from a broad range of natural and man-made fibres under standardised conditions. The dataset aims to facilitate reproducible research in fibre identification, classification, and spectral modelling by providing openly available, well-documented reference spectra suitable for chemometric and machine-learning applications.

## 3 Data Description

**Fig. 1:**
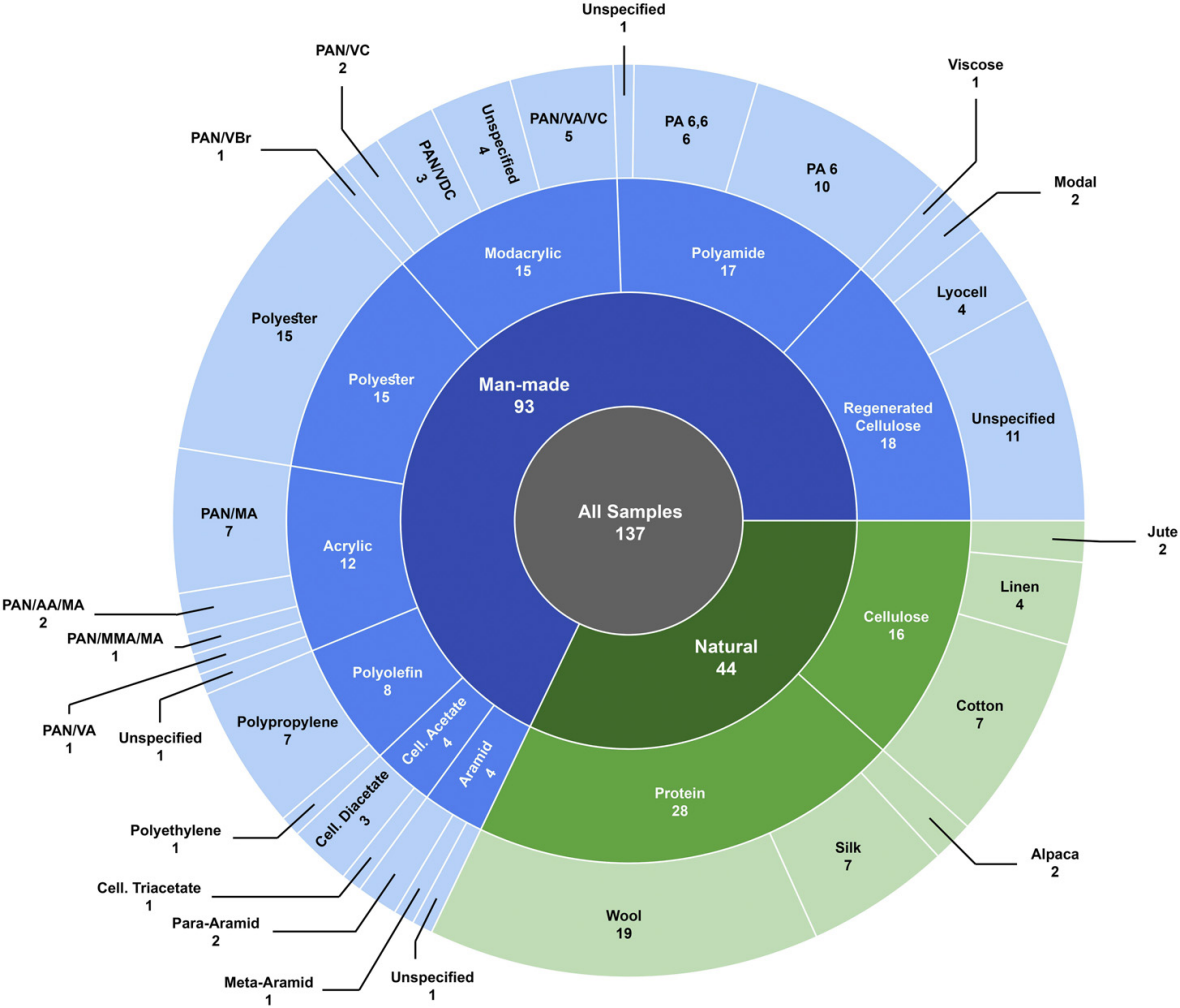
Sunburst diagram illustrating the composition of the dataset based on the number of fibre samples across fibre classes, types, and subtypes. The inner ring shows fibre origins (natural or man-made), the middle ring shows fibre types, and the outer ring represents the corresponding subtypes included in the collection. Segment sizes and the numeric labels shown within each segment both correspond to the number of samples assigned to each category.

The dataset, available on Mendeley Data [9], consists of ATR-FTIR spectra of textile fibre samples, organised into six main folders with consistent sample identifiers linking all files. The total number of spectra is 160, corresponding to 137 distinct fibre samples and covering 26 fibre subtypes distributed across natural and man-made textile categories. A summary overview of the dataset content is provided in Table 1, and an overview of the distribution of fibre classes, types and subtypes, based on the number of samples, represented in the collection is shown in Fig. 1.

**Table 1:** Summary of dataset content.

Folder	Format	Description	Files
01_raw_spectra/	.sp	Original instrument files	160
02_feature_matrix_raw/	.csv	Tabular feature matrix of unprocessed transmission spectra	1
03_feature_matrix_baseline_corrected/	.csv	Tabular feature matrix of baseline-corrected spectra	1
04_feature_matrix_baseline_corrected_average/	.csv	Tabular feature matrix of average baseline‐corrected spectra per subtype	1
05_feature_matrices_preprocessed/	.csv	Tabular feature matrices of pre-processed spectra prepared for chemometric and ML analyses	3
06_other_files/	.csv / .py	Sample metadata and Python preprocessing companion script	2

All tabular feature matrices (folders 02–05) share a consistent structure. Each row represents one measurement, with columns including a general spectrum identifier (Spectrum_ID), the sample of origin (Source_ID), the replica number (Replica) and categorical descriptors (Origin, Type, Subtype). These are followed by spectral variables corresponding to wavenumbers from 4000 cm⁻¹ to 550 cm⁻¹. The numeric values represent transmittance (0–1) for datasets in folders 02–04 and processed spectral intensities for the fully pre-processed dataset in folder 05. In addition, folder 06 contains a metadata table providing sample-level descriptors. Each row represents one material sample, including a unique sample identifier (Sample_ID), the source institution or supplier (Source), the reference collection from which the material was obtained (Collection), and categorical descriptors (Origin, Type, Subtype). Where available, additional descriptors are provided, including the manufacturer (Manufacturer), trade name (Trade_name), dye status (Dyed), and any further relevant notes (Additional_Details). These fields enable traceability of each spectrum to its original material source.

Folder and file structure:

1. 01_raw_spectra/ (160 SP files) — Contains original instrument output files in PerkinElmer .sp format. These files can be opened using software such as PerkinElmer Spectrum, Spectragryph, or other FT-IR data viewers supporting .sp files.
2. 02_feature_matrix_raw/ (1 CSV) — Tabular feature matrix of unprocessed transmission spectra. Each row corresponds to a measurement.
3. 03_feature_matrix_baseline_corrected/ (1 CSV) — Tabular feature matrix of baseline-corrected spectra generated using the asymmetric least-squares (ALS) method.
4. 04_feature_matrix_baseline_corrected_average/ (1 CSV) — Tabular feature matrix of average baseline-corrected spectra grouped by fibre subtype (e.g., wool, nylon 6).
5. 05_feature_matrix_preprocessed/ (1 CSV) — Tabular feature matrices of fully pre-processed spectra prepared using three alternative preprocessing pipelines: (i) conversion to absorbance, ALS baseline correction, and SNV scatter correction; (ii) conversion to absorbance, ALS correction, SNV, and Savitzky–Golay smoothing (15-point window, first derivative); and (iii) conversion to absorbance, ALS correction, SNV, and Savitzky–Golay smoothing (15-point window, second derivative).
6. 06_other_files (1 CSV, 1 Python script) — Contains a metadata table providing sample-level information and a Python companion script enabling users to apply the same preprocessing workflow to their own spectra.

**Fig. 2:**
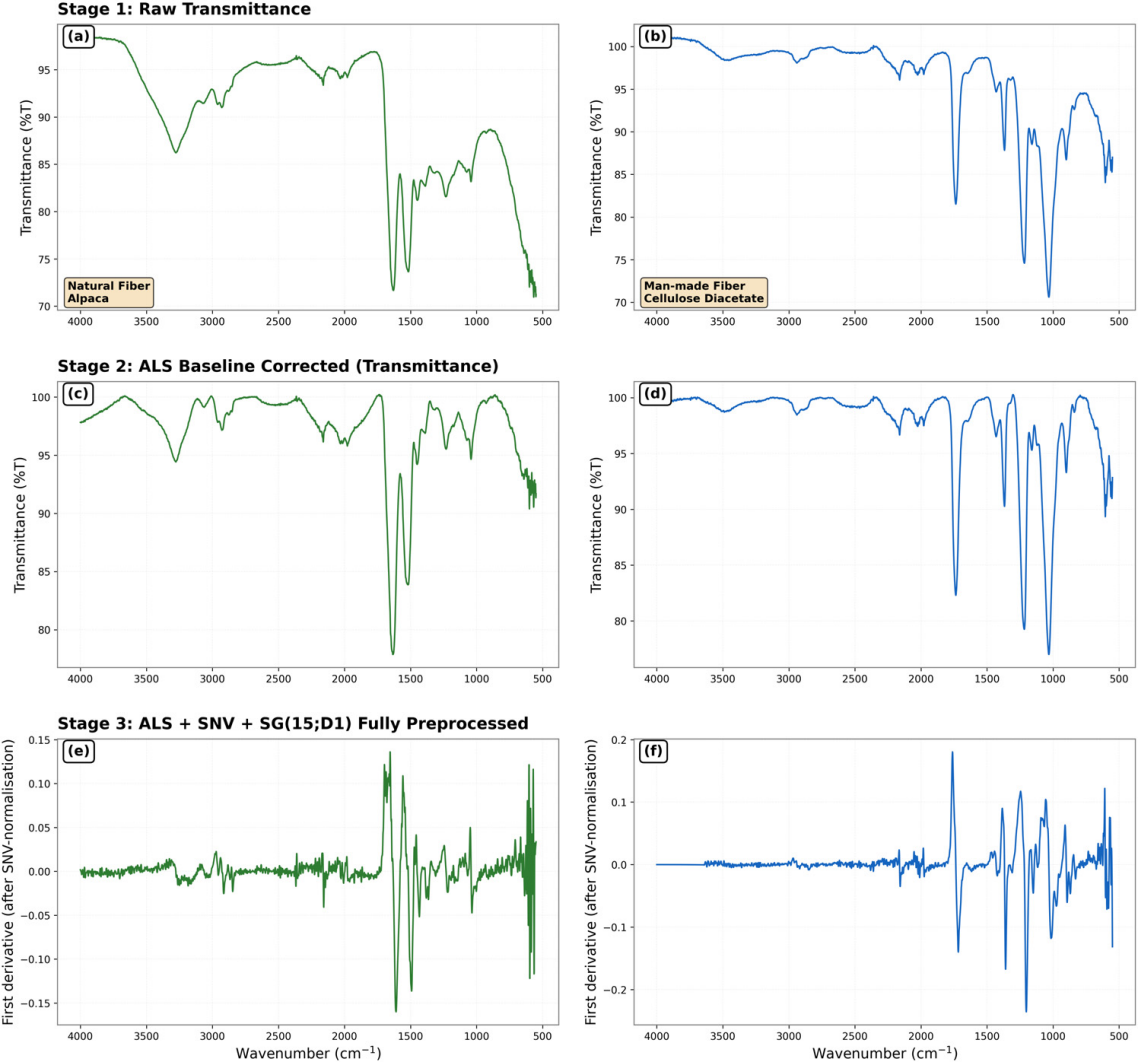
Representative ATR-FTIR spectra illustrating the effect of each processing stage for one natural fibre (left) and one man-made fibre (right). Rows show: (a-b) raw transmission spectra (%T); (c-d) baseline-corrected spectra after ALS correction (%T); and (e-f) fully pre-processed spectra following absorbance conversion, ALS correction, SNV, and Savitzky–Golay smoothing (15-point window, first derivative).

Representative examples of spectra at different processing stage (folders 02, 03 and 05) are illustrated in Fig. 2.

## 4 Experimental Design, Materials and Methods

**Fig. 3:**
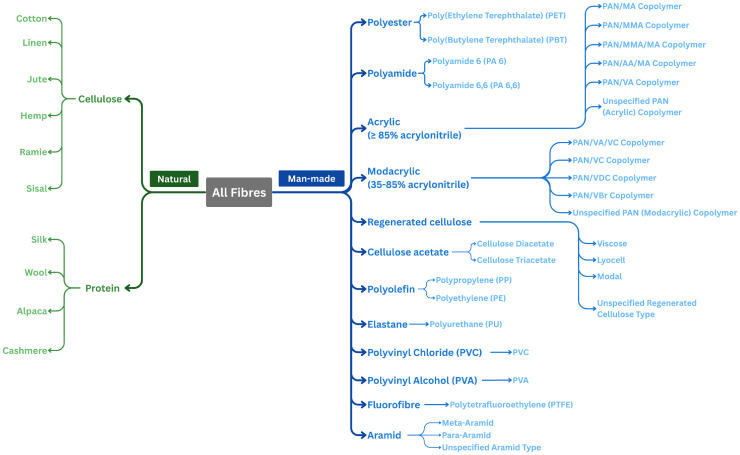
Hierarchical fibre classification scheme adopted for structuring the dataset, illustrating the organisation of textile fibres from origin (natural or man-made) to fibre type and subtype. This scheme was used to define the categorical descriptors reported in the dataset and to support consistent grouping of fibre materials.

**Sampling -** Textile fibre samples were collected from a range of verified sources to capture the diversity of materials encountered in clothing and household textiles. Samples were obtained from Microtrace LLC (Elgin, USA), specifically from their *Forensic Fiber Reference Collection* and *Arbidar Natural Fiber Collection*, and from the internal textile collections of the UNUSUWUL group at the University of the Arts London (London, UK) and the Bio-Couture group at Northumbria University (Newcastle, UK). All samples were supplied with confirmed identities, as provided by the respective suppliers. Only pure (non-blended) fibres were included. Samples were provided in various forms, including fibre tufts, yarns, and woven or knitted swatches. Variation in finishing and manufacturing treatments was intentionally retained to reflect real-world heterogeneity. The fibre classification scheme used in this study was developed based on common forensic practice and informed by the nomenclature principles outlined in ISO 2076 [10]. The resulting hierarchical structure is shown in Fig. 3.

**Spectral analysis -** Spectral measurements were carried out using a Frontier FT-IR spectrometer by PerkinElmer (Shelton, UK) equipped with a single-reflection diamond ATR accessory. Each sample was placed in direct contact with the ATR crystal and secured using the built-in pressure clamp. Spectra were acquired in transmission mode over the 4000–550 cm⁻¹ range at 4 cm⁻¹ spectral resolution with four co-added scans per acquisition. The applied contact pressure was typically set to 100 a.u., although this and the sample positioning were occasionally adjusted to optimise spectral quality. Adjustments were made to achieve a stable 100 % transmittance baseline (avoiding values above 100 %) and a total transmission range of at least 20 %, where possible. Spectra not meeting these quality conditions, or exhibiting excessive noise or visible artefacts attributable to poor crystal contact, were excluded from the final dataset. A background spectrum was collected before each analysis, and the ATR crystal was cleaned with acetone between samples to prevent contamination. Acetone was purchased by Sigma-Aldrich and was HPLC grade (>99.9 %) Multiple spectra were often acquired per sample, and one or several were retained. Replicates were collected to capture potential intra-sample heterogeneity (e.g., fibre morphology, surface treatments, or contact variability) and, where necessary, to improve representation of fibre subtypes with limited physical samples.

**Data processing -** All spectra were initially recorded as transmittance data (folder 02). Baseline correction was performed by first converting transmission spectra to absorbance, applying the asymmetric least-squares (ALS) algorithm, and subsequently converting the corrected spectra back to transmittance. This workflow was adopted because ALS operates by estimating and subtracting a baseline centred around zero intensity; therefore, conversion to absorbance (where the spectral baseline is approximately zero) is required to ensure mathematically appropriate correction. After baseline removal, spectra were reconverted to transmittance to preserve their conventional representation and facilitate visual comparison and reference use (folder 03). The same baseline-corrected spectra were then averaged by fibre subtype (e.g., wool, nylon 6) to produce a set of representative reference profiles (folder 04).

For use in chemometric and machine-learning applications, spectra were further processed using three alternative preprocessing pipelines, each provided as a separate tabular feature matrix (folder 05). In all pipelines, spectra were converted to absorbance to linearise intensity relationships, baseline-corrected by ALS to mitigate baseline drift, and normalised using standard normal variate (SNV) to reduce multiplicative scatter effects and variability due to fibre–crystal contact and effective pathlength. Two pipelines additionally applied Savitzky–Golay filtering to compute first- or second-derivative spectra (15-point window), which can enhance subtle spectral features and improve discrimination between chemically similar fibres by reducing broad background contributions and resolving overlapping bands, albeit at the cost of increased noise sensitivity.

These alternative preprocessing strategies are provided to support different classification approaches and modelling objectives. Their performance in supervised classification tasks is summarised in Table 2, as evaluated using the classification procedures described below.

**Table 2:** Cross-validated classification accuracies (%) for different spectral preprocessing pipelines evaluated using support vector machine (SVM) and random forest (RF) classifiers. Results are reported for binary classification by fibre origin (natural vs man-made) and multi-class classification by fibre type, based on 5-fold cross-validation.

Pipeline	Accuracy [%]			
	SVM model		RF model	
	Binary	Multi-class	Binary	Multi-class
*ALS + SNV*	93.12	94.38	85.62	95.00
*ALS + SNV + SG(15;D1)*	86.25	88.75	94.38	88.12
*ALS + SNV + SG(15;D2)*	72.50	75.62	83.12	80.62

**Classification modelling and preprocessing evaluation -** To assess the suitability of the different spectral preprocessing pipelines described above for classification tasks, supervised classification models were trained using support vector machines (SVM) and random forests (RF). Both binary and multi-class classification problems were considered, where binary classification corresponded to discrimination by fibre origin (natural vs man-made) and multi-class classification corresponded to discrimination by fibre type. Prior to modelling, the preprocessed spectral data were subjected to dimensionality reduction using principal component analysis (PCA) to summarise spectral variability and reduce feature dimensionality. The first 10 principal components were retained, as they accounted for at least 90 % of the total variance across all preprocessing configurations, and were subsequently used as input variables for SVM and RF classification. Model hyperparameters were tuned using standard resampling procedures, and performance was evaluated using 5-fold cross-validation. Classification accuracy was calculated as the proportion of correctly classified spectra averaged across cross-validation folds (Table 2).

For reproducibility, all preprocessing parameters (including ALS and Savitzky–Golay settings) and modelling hyperparameters are summarised in Table 3.

**Table 3:** Summary of preprocessing and modelling parameters used for baseline correction, spectral filtering, and supervised classification in this study.

*Category*	*Parameter*	*Value*	*Description*
*ALS baseline correction*	λ (lambda)	1 × 10⁶	Smoothness parameter
	p	0.001	Asymmetry parameter
	n_iter	10	Number of iterations
*Savitzky–Golay filter*	window_length	15	Filter window size
	polyorder	3	Polynomial order
	deriv	1 or 2	Derivative order (pipelines 2 and 3)
*SVM classifier*	kernel	rbf	Radial basis function kernel
	C	10	Regularisation parameter
	gamma	scale	Kernel coefficient
*RF classifier*	n_estimators	200	Number of trees
	max_features	sqrt	Number of features per split

**Software -** All spectra were acquired using Spectrum software v10.4.00 by PerkinElmer. Data processing was performed in Python v3.12.0 [11] using the libraries Numpy [12], Pandas [13], SciPy [14] and Matplotlib [15] for baseline correction, spectral conversion, filtering, and data handling. All scripts used for preprocessing, model training, and performance evaluation are openly available in a GitHub repository [16]. A companion script is also included in the repository to enable users to apply the same correction and preprocessing pipeline to their own spectra, ensuring consistency with the procedures used to generate the processed datasets presented here.

## Limitations

The dataset currently includes 26 fibre subtypes, covering the most common natural and man-made materials encountered in clothing and household textiles. Data collection is ongoing, and additional fibre subtypes will be incorporated over time. Although efforts were made to include variability in sample form and manufacturing treatment, the representation of some fibre categories remains more limited than others. Future updates will also expand the dataset by adding further spectra for previously sampled materials. No other major limitations were identified.

## Ethics Statement

The current work does not involve human subjects, animal experiments, or any data collected from social media platforms.

## CRediT authorship contribution statement

**Reeha Parkar:** Conceptualization, Methodology, Software, Formal analysis, Investigation, Data curation, Writing – original draft, Visualization. **Angelica Jain:** Investigation, Data curation. **Miranda Prendergast-Miller:** Resources, Data curation, Writing – original draft. **Thomas Stanton:** Resources, Data curation, Writing – original draft. **Kelly J. Sheridan:** Resources, Data curation, Writing – original draft. **Matteo D. Gallidabino:** Conceptualization, Methodology, Resources, Data curation, Writing – original draft, Supervision, Project administration, Funding acquisition.

## Data Availability

(Mendeley Data).A dataset of infrared (ATR-FTIR) spectra for textile fibres of natural and man-made origin (Original data)

## References

[1] Stanton T., Johnson M., Nathanail P., MacNaughtan W., Gomes R.L. (2019). Freshwater and airborne textile fibre populations are dominated by ‘natural’, not microplastic, fibres. Sci. Total Environ..

[2] Napper I.E., Parker-Jurd F.N.F., Wright S.L., Thompson R.C. (2023). Examining the release of synthetic microfibres to the environment via two major pathways: atmospheric deposition and treated wastewater effluent. Sci. Total Environ..

[3] Jones J., Johansson S. (2023). A population study of textile fibres on the seats at three public venues. Forensic Sci. Int..

[4] Allan H.K., Fricker A.E., Hsieh Y.-L. (2024). A trace fiber population study on upholstered chairs in a military environment. Forensic Sci. Int..

[5] Meleiro P.P., García-Ruiz C. (2016). Spectroscopic techniques for the forensic analysis of textile fibers. Appl. Spectrosc. Rev..

[6] Peets P., Leito I., Pelt J., Vahur S. (2017). Identification and classification of textile fibres using ATR-FT-IR spectroscopy with chemometric methods. Spectrochim. Acta A: Mol. Biomol. Spectrosc..

[7] Sharma V., Mahara M., Sharma A. (2024). On the textile fibre’s analysis for forensics, utilizing FTIR spectroscopy and machine learning methods. Forensic Chem..

[8] da Cruz Santos E., Silva A.A.B., Faria R.R.A., de Almeida Rizzutto M., Rodrigues P.H.S., Baruque-Ramos J. (2024). Raw cellulosic fibers: characterization and classification by FTIR-ATR spectroscopy and multivariate analysis (PCA and LDA). Mater. Circ. Econ..

[9] Parkar R., Jain A., Prendergast-Miller M., Stanton T., Sheridan K., Gallidabino M. (2026). A dataset of infrared (ATR-FTIR) spectra for textile fibres of natural and man-made origin. Mendeley Data.

[10] International Organization for Standardization, ISO 2076:2021 textiles — Man-made fibres — Generic names, ISO, Geneva, Switzerland, 2021. https://www.iso.org/standard/79685.html. [Accessed: Feb. 24, 2026].

[11] G. Van Rossum, F.L. Drake, Python 3 Reference manual, CreateSpace, Scotts Valley, CA, 2009. https://dl.acm.org/doi/book/10.5555/1593511. [Accessed: Feb. 24, 2026].

[12] Harris C.R., Millman K.J., van der Walt S.J., Gommers R., Virtanen P., Cournapeau D., Wieser E., Taylor J., Berg S., Smith N.J., Kern R., Picus M., Hoyer S., van Kerkwijk M.H., Brett M., Haldane A., del Río J.F., Wiebe M., Peterson P., Gérard-Marchant P., Sheppard K., Reddy T., Weckesser W., Abbasi H., Gohlke C., Oliphant T.E. (2020). Array programming with NumPy. Nature.

[13] McKinney W. (2010). Proc. 9th Python Sci. Conf..

[14] Virtanen P., Gommers R., Oliphant T.E., Haberland M., Reddy T., Cournapeau D., Burovski E., Peterson P., Weckesser W., Bright J., van der Walt S.J., Brett M., Wilson J., Millman K.J., Mayorov N., Nelson A.R.J., Jones E., Kern R., Larson E., Carey C.J., Polat İ., Feng Y., Moore E.W., VanderPlas J., Laxalde D., Perktold J., Cimrman R., Henriksen I., Quintero E.A., Harris C.R., Archibald A.M., Ribeiro A.H., Pedregosa F., van Mulbregt P., Vijaykumar A., Bardelli A.P., Rothberg A., Hilboll A., Kloeckner A., Scopatz A., Lee A., Rokem A., Woods C.N., Fulton C., Masson C., Häggström C., Fitzgerald C., Nicholson D.A., Hagen D.R., Pasechnik D.V., Olivetti E., Martin E., Wieser E., Silva F., Lenders F., Wilhelm F., Young G., Price G.A., Ingold G.-L., Allen G.E., Lee G.R., Audren H., Probst I., Dietrich J.P., Silterra J., Webber J.T., Slavič J., Nothman J., Buchner J., Kulick J., Schönberger J.L., de Miranda Cardoso J.V., Reimer J., Harrington J., Rodríguez J.L.C., Nunez-Iglesias J., Kuczynski J., Tritz K., Thoma M., Newville M., Kümmerer M., Bolingbroke M., Tartre M., Pak M., Smith N.J., Nowaczyk N., Shebanov N., Pavlyk O., Brodtkorb P.A., Lee P., McGibbon R.T., Feldbauer R., Lewis S., Tygier S., Sievert S., Vigna S., Peterson S., More S., Pudlik T., Oshima T., Pingel T.J., Robitaille T.P., Spura T., Jones T.R., Cera T., Leslie T., Zito T., Krauss T., Upadhyay U., Halchenko Y.O., Vázquez-Baeza Y. (2020). SciPy 1.0: fundamental algorithms for scientific computing in Python. Nat. Methods.

[15] Hunter J.D. (2007). Matplotlib: a 2D graphics environment. Comput. Sci. Eng..

[16] Parkar R., Gallidabino M. (2025). FasTEX-KCL (Version 1). GitHub.

